# Influence of Intratumor Heterogeneity on the Predictivity of MGMT Gene Promoter Methylation Status in Glioblastoma

**DOI:** 10.3389/fonc.2020.533000

**Published:** 2020-10-20

**Authors:** Giovanni Brigliadori, Giulia Goffredo, Daniela Bartolini, Luigino Tosatto, Lorena Gurrieri, Laura Mercatali, Toni Ibrahim

**Affiliations:** ^1^Bioscience Laboratory, Istituto Scientifico Romagnolo per lo Studio e la Cura dei Tumori (IRST) IRCCS, Meldola, Italy; ^2^Pathological Anatomy Unit, Bufalini Hospital, Cesena, Italy; ^3^Neurosurgery Unit, Bufalini Hospital, Cesena, Italy; ^4^Osteoncology and Rare Tumors Center, Istituto Scientifico Romagnolo per lo Studio e la Cura dei Tumori (IRST) IRCCS, Meldola, Italy

**Keywords:** temozolamide, MGMT methylation, intratumor heterogeneity, predictivity, glioblastoma

## Abstract

Glioblastoma is the most aggressive tumor of the central nervous system. Prognosis is poor, even in the presence of a methylated state of MGMT gene promoter, which represents the biomarker with the highest prognostic/predictive value for the standard treatment of patients. Among patients with a methylated MGMT status, we identified an intermediate range of methylation above the standard 9% cut-off (gray zone) in which the predictive strength of the marker was lost. In an effort to improve the evaluation of the biomarker in clinical decision-making, we are carrying out a retrospective study, performing an in-depth analysis of samples used for diagnosis to understand how molecular heterogeneity, a hallmark of glioblastoma, impacts the evaluation of MGMT gene promoter methylation. Preliminary data from samples belonging to the “gray zone” tend to confirm the hypothesis of a mismatch between methylation values used for clinical decision-making and those included in our in-depth analysis. Confirmation of these data would help to better define the predictive power of MGMT promoter methylation status and greatly facilitate clinical decision-making.

Among brain tumors, glioblastoma (grade IV according to World Health Organization) is the most aggressive form of disease, with an average survival ranging from 12 to 15 months (1, 2). Currently, only a small number of molecular markers are recognized in brain diseases compared to other cancers. One of the molecular markers with the highest prognostic/predictive impact in glioblastoma is the methylation status of the promoter of the O_6_-methylguanine DNA methyltransferase (MGMT) gene, which encodes for an enzyme involved in the DNA repair system. Standard treatment for glioblastoma is the “Stupp protocol” (3), comprising radiotherapy and chemotherapy with the alkylating agent temozolomide (TMZ). When the MGMT promoter is in a “methylated” state, a better response to the treatment is expected.

The main issues relating to the evaluation of the degree of methylation of the MGMT promoter are as follows:

- There is no cutoff value that uniquely discriminates between methylated and unmethylated states. Using the quantitative pyrosequencing method, the current reference value is 9% (4).
- A precise evaluation of the prognostic/predictive value of the degree of methylation is still a matter of debate.

We carried out a retrospective study aimed at defining a methylation cutoff value, identifying a value of 30% methylation as discriminant between the methylated and unmethylated state (5). Of note, we found that patients who underwent the same type of surgery (radical or non-radical) and had a MGMT methylation value ranging from 10 to 29% showed a poorer overall survival (OS) than those with unmethylated MGMT (9.8 vs. 19.5 months, respectively). Starting from this observation, we decided to perform an in-depth evaluation of this subset of patients in whom the predictive power of the marker is lost, calling the methylation range (10–29%) in question the *gray zone*.

Given the well-known molecular heterogeneity of the tumor, we hypothesize that the mismatch with the predictive value of the marker could be due to misinterpretation of the methylation status. Several studies are currently underway to investigate the clinical/biological impact of this tumor characteristic, which includes MGMT promoter methylation, and differ mainly in their approach to the problem:

- Primary cell culture models isolated from neoplastic lesions (6)
- Molecular analysis on bioptic sections of different areas of the tumor mass (7)
- Assessment of methylation variability of the individual CpG islands within a methylation profile (8).

Although all of these approaches can improve our understanding of the molecular heterogeneity of glioblastoma, their impact on diagnostic decision-making requires further investigation.

We decided to evaluate intratumor heterogeneity in single formalin-fixed paraffin-embedded (FFPE) tissue samples used for diagnosis, using identical subsections of each sample to improve the analysis and to obtain a more accurate evaluation of the methylation status of the MGMT promoter. To this end, we are carrying out a retrospective study on a set of samples from 120 patients with a follow-up of at least 2 years, stratified into four groups of 30 patients each (Figure 1A):

a) Non-methylated (0–9%)
b) Low methylated (10–17%)
c) Medium methylated (18–29%)
d) Highly methylated (30–100%).

**Figure 1:**
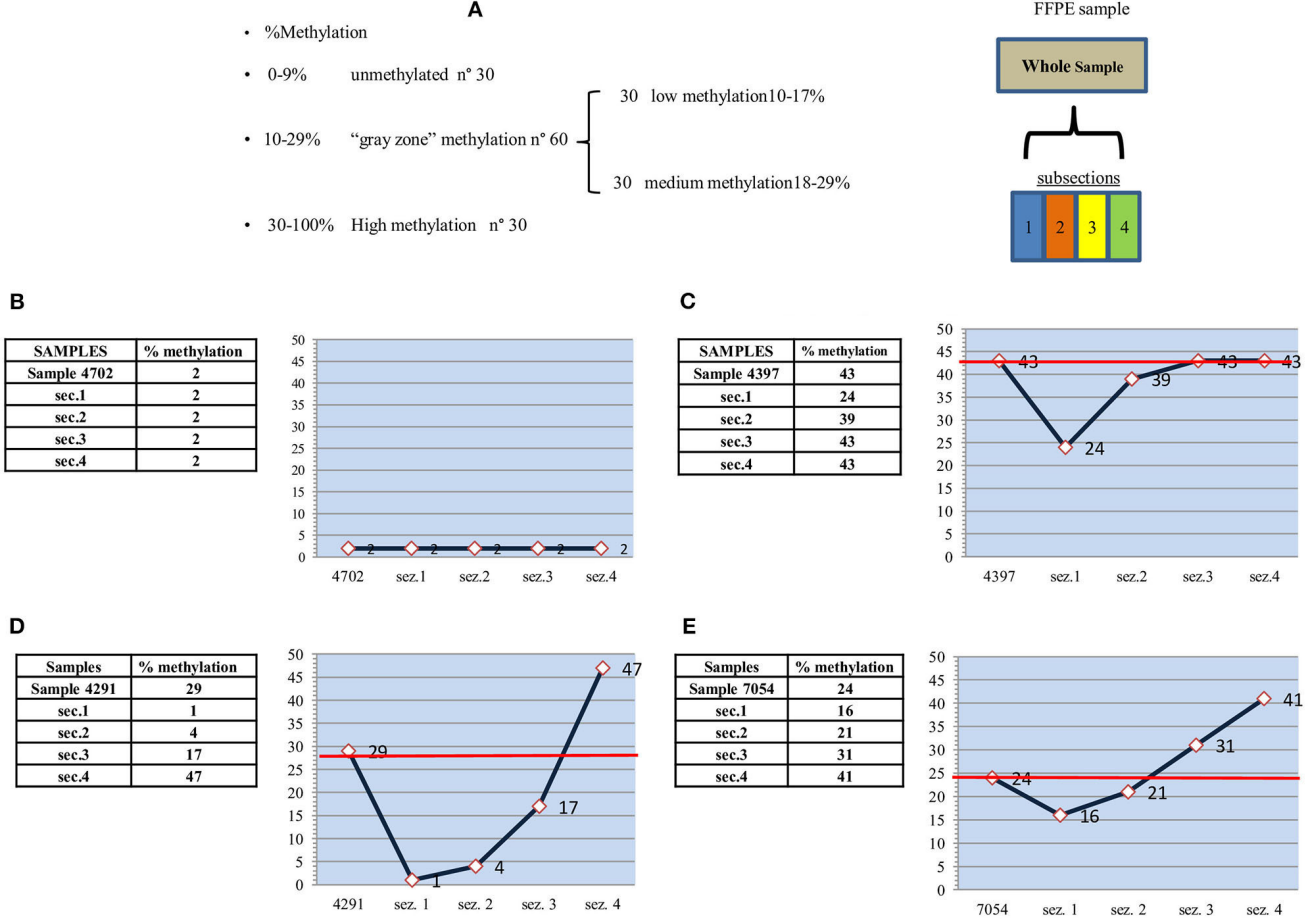
Study design, unmethylated, highly and medium methylated samples. **(A)** Study design: MGMT promoter methylation analysis. **(B)** “unmethylated” sample-2% - and subsections. **(C)** “highly methylated” sample-43% - and subsections. **(D)** “medium methylated” sample-29% - and subsections. **(E)** “medium methylated” sample-24% - and subsections.

Two FFPE sections are used for each sample. The DNA of the entire lesion is extracted from one section, whereas the other section is further divided into four subsections, each subjected separately to DNA extraction. Given the impossibility of using a histological basis to subdivide samples, sections are arbitrarily selected, and their homogeneity evaluated by quantifying the amount of DNA obtained in an equal volume of elution buffer.

In a preliminary analysis, we found that the degree of MGMT promoter methylation was fairly similar in some cases, whereas in others it showed considerable variability, with high values in one section and much lower values in others. According to our study design, it is reasonable to assume that, if the methylation value of the entire sample is >30%, there will be a homogenously high methylation in the subsections. Similarly, a uniformly low methylation is expected in subsections when the overall methylation value is <9%. The greatest heterogeneity is expected when the average methylation of the entire sample falls within the gray zone (10–17% and 18–29%).

## Preliminary Results

Ten samples were analyzed. Eight samples were within the gray zone: six showing medium methylation (21, 22, 23, 24, and 29%) and two low methylation (each 15%). One sample was in unmethylated state (2%), and one was highly methylated (43%).

As expected, the highly methylated (>30%) and unmethylated (<9%) samples (Figure 1) showed subsection methylation values within the considered range. The unmethylated sample (Figure 1B) fell into the narrowest range (0–9%) and thus showed the greatest uniformity. The sample with an average 43% methylation (Figure 1C) had three out of four sections with methylation >30%, and only one section with a lower value (24%), which was still fairly high with respect to the standard cutoff of 9%. The red line indicates the average methylation of the entire sample.

**Figure 2:**
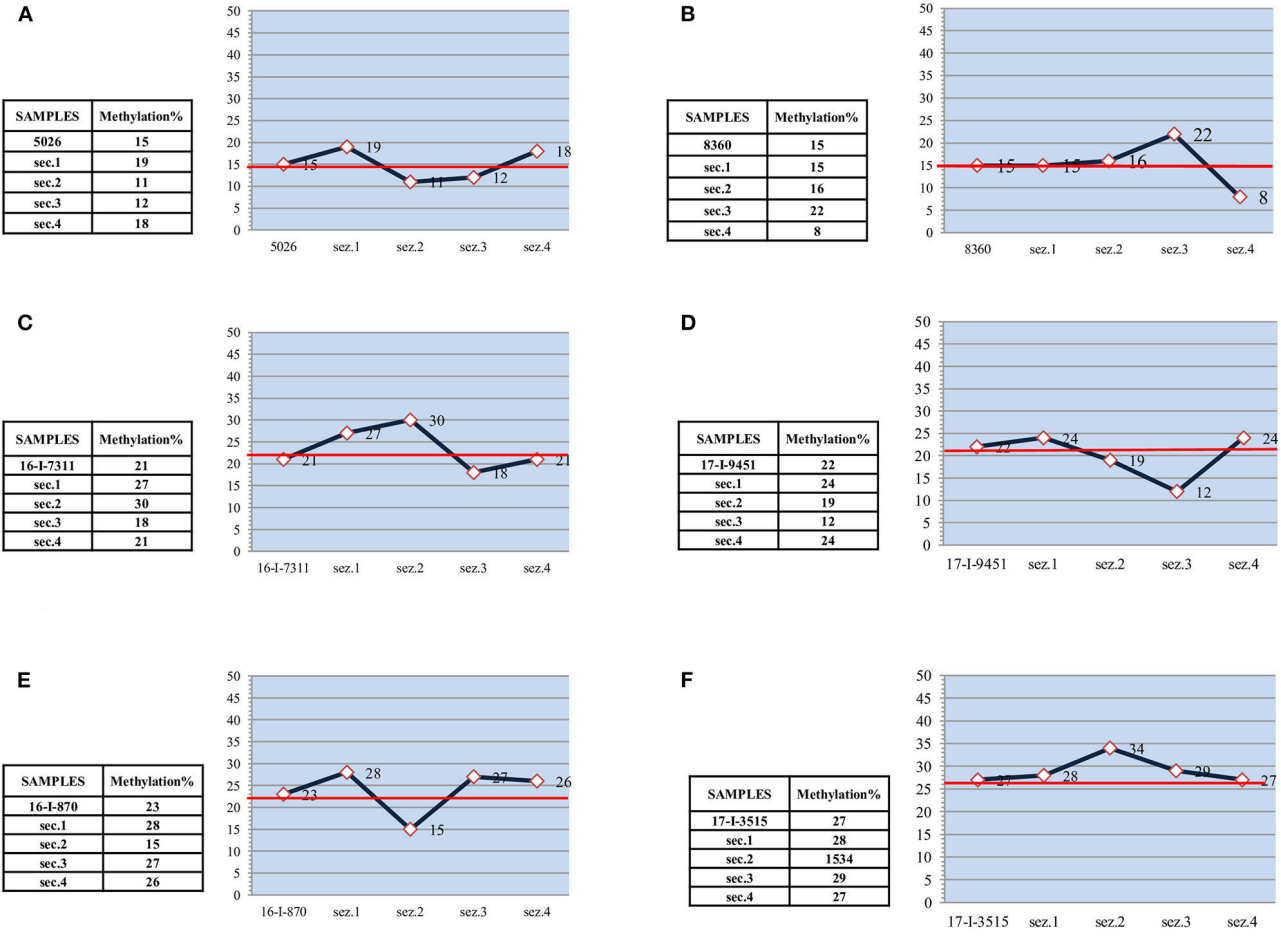
Low and medium methylated samples. **(A**) “low methylated” sample-15% - and subsections. **(B)** “low methylated” sample-15% - and subsections. **(C)** “medium methylated” sample-21% - and subsections. **(D)** “medium methylated” sample-22% - and subsections. **(E)** “medium methylated” sample-23% - and subsections. **(F)** “medium methylated” sample-27% - and subsections.

Considerable variability in the methylation values of single sections was observed in samples belonging to the gray zone. This variability was also present in low methylation samples (Figures 2A,B), in which sections showed differences of at least three percentage points with respect to the mean value of the entire sample. In one case (Figure 2B, sample section 4), a value fell into the unmethylated range, which, given the narrow range of values (10–17%), would seem to confirm molecular heterogeneity.

Differences between single sections were more marked in samples with medium methylation. In particular, mean methylation values of 29 and 24% (Figures 1D,E) could lead to an incorrect evaluation of methylation status. In the former (Figure 1D), there was high methylation (47%) in one subsection and low methylation (17%) in the second, whereas the remaining subsections were unmethylated (4 and 1%). Thus, compared to the methylation value that would place the sample in the methylated category, 50% of the sample was unmethylated, and 25% was low methylated. In the latter sample (Figure 1E), in contrast to the previous case, 50% of sample subsections showed higher than average methylation values (41 and 31% vs. 24%). Thus, paradoxically, the apparently less methylated sample was actually more methylated. Considering the two samples, the seemingly more highly methylated one was, in fact, the least methylated.

In the other medium methylated samples (Figures 2C–F), the differences between the subsections and the entire sample were less remarkable. However, there was always at least one section in each sample showing a methylation value that definitely deviates from the average.

## Discussion

Although great efforts have been made to improve the outcome of patients with glioblastoma, it remains the leading cause of death among brain tumors, with a dismal prognosis (2). Surgery is the mainstay of treatment, and the Stupp protocol (radiotherapy and chemotherapy with TMZ) (3) represents the only postsurgery treatment obtaining a benefit in either progression-free survival or OS (9–12). The efficacy of TMZ is mainly related to MGMT promoter methylation status, which represents the only prognostic/predictive marker for these patients. However, there are many unanswered questions about the role of MGMT methylation status in patient outcome, its cutoff threshold, and predictive strength. Our previous investigation of these issues led us to propose a 30% methylation cutoff (4). In the same study we identified a subset of patients in which the predictivity of the marker was lost, calling this MGMT methylation range (10–29%) the “gray zone.” We hypothesize that this loss of predictivity could be influenced by the molecular heterogeneity of the disease. We are therefore performing a retrospective study to investigate the correlation between the intratumor heterogeneity of MGMT promoter methylation and patient outcome. Our preliminary data appear to confirm the well-known histological heterogeneity of the disease at the molecular level and indicate the need for a more in-depth evaluation of samples belonging to the gray zone.

With regard to the prognostic/predictive value of the marker, we hypothesize that molecular heterogeneity may influence its clinical evaluation, especially in cases that fall within the gray zone. Our preliminary data appear to confirm this because gray zone samples showed an internal methylation distribution that differed significantly from the mean value used for the diagnostic referral. For example, in one case with a relatively high mean methylation (29%) for the entire sample, the value was mainly due to a single section with a very high methylation status (47%), whereas much of the sample showed low (17%) or no methylation (Figure 1D). Conversely, a moderately high mean methylation (24%) in another sample had a methylation distribution in which two of the four sections showed values higher than the entire sample (31 and 41%) (Figure 1E). Consequently, most of the sample was more highly methylated than the mean value used for clinical decision-making. Moreover, intratumor heterogeneity was well-represented, albeit to a lower degree, in all the other samples belonging to the gray zone.

## Conclusions

The small number of samples analyzed is probably the most important limitation of the present study. Despite this, we believe that the heterogeneity found in MGMT promoter methylation values provides sufficient evidence to warrant further investigation. We intend to complete the study with data on patient follow-up and analysis of all cases and will carry out a more in-depth analysis of the samples in which MGMT promoter methylation falls into the gray zone to enhance the prognostic/predictive capacity of the marker and facilitate treatment decision-making.

## Data Availability Statement

The raw data supporting the conclusions of this article will be made available by the authors, without undue reservation, to any qualified researcher.

## Ethics Statement

The protocol was approved by the IRST Medical Scientific Committee, approval no. 5870/5.3, and performed according to Good Clinical Practice standards and the Declaration of Helsinki. All patients gave written informed consent to take part in the study.

## Author Contributions

GB and GG performed the analyses and drafted the manuscript. DB prepared and selected the FFPE samples. LT performed the surgery. LG selected patients suitable for the study. LM reviewed the manuscript for important intellectual content. TI selected patients and reviewed the manuscript for important intellectual content. All authors contributed in equal part to the study design, read and approved the final version of the manuscript for publication.

## Conflict of Interest

The authors declare that the research was conducted in the absence of any commercial or financial relationships that could be construed as a potential conflict of interest.
